# The Comprehensive Adaptive Multisite Prevention of University Student Suicide Trial: Protocol for a Randomized Controlled Trial

**DOI:** 10.2196/68441

**Published:** 2025-04-22

**Authors:** Kyla Blalock, Jacqueline Pistorello, Shireen L Rizvi, John R Seeley, Francesca Kassing, James Sinclair, Linda A Oshin, Robert J Gallop, Cassidy M Fry, Ted Snyderman, David A Jobes, Jennifer Crumlish, Hannah R Krall, Susan Stadelman, Filiz Gözenman-Sapin, Kate Davies, David Steele, David B Goldston, Scott N Compton

**Affiliations:** 1 Duke University Durham, NC United States; 2 University of Nevada, Reno Reno, NV United States; 3 Rutgers, The State University of New Jersey New Brunswick, NJ United States; 4 University of Oregon Eugene, OR United States; 5 University of Arkansas at Fayetteville Fayetteville, AR United States; 6 West Chester University West Chester, PA United States; 7 Catholic University of America Washington D.C., DC United States; 8 Lurie Children's Hospital Chicago United States

**Keywords:** suicide, adaptive treatment strategies, Collaborative Assessment and Management of Suicidality, CAMS, dialectical behavior therapy, DBT, university students

## Abstract

**Background:**

Suicidal ideation is increasing among university students. Despite growing demand for services, university counseling centers (UCCs) face limited resources to meet the complex needs of students who are suicidal.

**Objective:**

The Comprehensive Adaptive Multisite Prevention of University Student Suicide (CAMPUS) Trial evaluates 4 treatment sequences within UCCs to develop evidence-based treatment guidelines.

**Methods:**

The CAMPUS Trial consists of a feasibility study followed by a sequential multiple-assignment randomized trial (SMART). The original CAMPUS protocol was modified during the COVID-19 pandemic to accommodate new UCC tele–mental health services, including remote treatment, assessments, and monitoring. A smaller-scale feasibility study was conducted to (1) evaluate implementation of hybrid telehealth and in-person interventions and (2) fine-tune online procedures. Following the feasibility study, university students (aged 18-25 years) seeking UCC services with moderate to severe suicidal ideation will enroll in the CAMPUS Trial. Student participants are randomly assigned to 1 of 4 treatment sequences with 2 stages of intervention. In stage 1, students receive 4 to 6 weeks of either (1) a suicide-focused treatment—Collaborative Assessment and Management of Suicidality—or (2) enhanced treatment as usual. Treatment responders enter the maintenance phase. In stage 2, nonresponders are rerandomized for an additional 1 to 8 weeks of (1) Collaborative Assessment and Management of Suicidality or (2) an intensive skills-based treatment—dialectical behavior therapy for UCC settings. UCC counselors will enroll in the CAMPUS Trial to complete measures about their experience working with students who are suicidal. CAMPUS Trial administration includes representation from all sites to facilitate cross-site coordination and an advisory board of stakeholders from all UCCs to facilitate treatment implementation.

**Results:**

Student participant recruitment began on October 25, 2022, and ended on May 16, 2024. As of November 2024, data collection for the SMART was ongoing with active study participants. Data collection was completed in November 2024, and as of April 2025, data analysis is underway. Full results will be available in 2025.

**Conclusions:**

The CAMPUS Trial offers a model for future SMARTs for the treatment of suicidal thoughts or behaviors (or both) across various settings. The results will inform treatment guidelines for students presenting with suicidality at UCCs.

**Trial Registration:**

ClinicalTrials.gov NCT04707066; http://clinicaltrials.gov/ct2/show/NCT04707066

**International Registered Report Identifier (IRRID):**

DERR1-10.2196/68441

## Introduction

### Background

Among university students, suicide is the second leading cause of death [1]; 6% of university students report suicide attempts (SAs) in their lifetime, and 2.4% report attempting suicide in the last year alone [2,3]. Suicidal ideation (SI) is even more frequent. Approximately 1 in 4 students report a history of SI, including 14% in the last year [4,5]. These numbers are climbing among university students—paralleling an increase in suicide rates among individuals aged 10 to 24 years from 2007 to 2021 [6]. University counseling centers (UCCs) are the front line for mental health services for a growing number of students experiencing SI [7,8]. However, UCCs face limited resources, higher demand for services, and increasingly complex cases [9,10], with more than half reporting that waitlists develop within a few weeks of a new academic year and last throughout the term [11].

Managing students presenting with SI in UCCs can be challenging due to heterogeneity in presentations. Some university students struggling with SI respond rapidly to treatment, whereas others require considerably more time and resources [12]. Despite aspiring to provide brief therapy (5-6 sessions) [7], UCCs often provide ≥20 sessions to a segment of their student population [13]. In fact, 20% of students have been shown to use 50% of counseling resources [13], with highly distressed students needing more resources [12]. Suicidal thoughts or behaviors (STB; or both) are likely a key driver of this pattern of use as SI has become a primary presentation at UCCs [14], with over one-third of treatment-seeking students reporting serious SI in the last year [13]. Students with persistent SI place a significant strain on UCC resources and use 20% to 30% more services than other students [15]. Use of UCC resources devoted to *rapid access* services, which guarantee an appointment within 1 to 5 days for students in crisis, has increased by 28% [16]. Despite this need, there is currently no *gold standard* approach for treating students with SI in UCCs. Due to the varying levels of severity and treatment needs among university students struggling with STB, UCCs would benefit from evidence-based guidelines to inform clinical decision-making.

The Comprehensive Adaptive Multisite Prevention of University Student Suicide (CAMPUS) Trial is a National Institute of Mental Health (NIMH)–funded multisite sequential multiple-assignment randomized trial (SMART). The aim of this study is to evaluate the relative effectiveness of 4 adaptive treatment strategies (ATSs) for university students who report SI when seeking services for a new course of care at a UCC. The CAMPUS Trial ATSs follow a stepped-care model of treatment delivery, with those not responding to an initial course of brief therapeutic care being offered additional treatment of longer duration or greater intensity. The implementation of these ATSs will be monitored to better understand the acceptability, appropriateness, feasibility, fidelity, and costs related to suicide-focused treatment within UCCs. This hybrid effectiveness-implementation approach combines components of clinical effectiveness and implementation research, facilitating the future translation of empirically validated treatments within the UCC context.

### Rationale for Study Design

#### What Is a SMART?

A SMART is a type of factorial design used to develop and optimize adaptive interventions by studying treatments in a sequential manner [17-19]. In a SMART, participants can receive multiple interventions over time depending on their response to previous treatments and may be randomized more than once. For example, if a participant does not sufficiently respond to an initial course of care that they were initially randomized to receive, they might be rerandomized to one of several second-line treatments. The number of stages and the specific treatments received by a participant depend on their response at each stage, guided by one or more tailoring variables that informs clinical decision-making. The ultimate goal of SMARTs is to develop empirically supported clinical guidelines for providing treatment and modifying the course of care based on an individual’s baseline characteristics and other time-varying outcomes, such as treatment response and compliance [17-19]. ATSs are vital for personalizing the provision of care to improve outcomes and ensure that interventions are responsive to the changing needs of individuals.

#### Rationale for the Use of a SMART Design to Treat STB Within UCCs

ATSs are recommended in situations in which (1) patients vary in their response to treatment, (2) the effectiveness of an intervention changes over time due to fluctuating symptoms, (3) comorbidities make treatment more complex, or (4) there is a high probability of relapse [18]. All of these conditions apply when treating suicidal university students. UCCs will likely benefit from data on how to tailor treatments to individual students to better manage their finite resources and optimize outcomes. A similar pilot SMART was previously conducted and served as the foundation for this study [20]. However, that pilot study had several limitations: it had a small sample size; was conducted at a single UCC; relied solely on in-person assessments and treatments; and evaluated ATSs of considerably longer duration (up to 24 weeks), which was not conducive to wide dissemination and adoption across various UCC contexts. The CAMPUS Trial design addressed these shortcomings.

#### Overview of the ATSs Evaluated in the CAMPUS Trial

The goal of the CAMPUS Trial is to evaluate sequences of treatments for use in UCCs to treat a wide range of university students presenting with STB. It follows a stepped-care model that begins with shorter and less resource-intensive treatments [21,22] and continues with more intensive treatments for students who do not respond sufficiently to the initial course of care. To improve dissemination of the ATSs, the duration of each treatment stage is kept brief to match the practices of UCCs (5-6 sessions on average) [13]. The schedule of enrollment, interventions, and assessments can be found in Table 1.

The CAMPUS Trial aims to identify sequences of care across 2 stages of treatment, with clear decision points for optimal outcomes. Stage 1 evaluates whether starting with a suicide-focused treatment (ie, Collaborative Assessment and Management of Suicidality [CAMS]) is superior to traditional, non–suicide-focused psychotherapies commonly provided in UCCs (ie, enhanced treatment as usual [E-TAU]). Stage 2 assesses whether switching to a more intensive skills-based treatment (ie, counseling center dialectical behavior therapy [CC-DBT]) is superior to continuing CAMS among insufficient responders to stage 1 CAMS and whether CC-DBT is superior to CAMS among insufficient responders to stage 1 E-TAU. Both CAMS and dialectical behavior therapy (DBT) have been shown to reduce SI, suicidal behavior, or both in university students [20,23] and have already been adopted in some UCCs [14,24]. The ATSs evaluated in the CAMPUS Trial align closely with the Zero Suicide policy initiative [25,26] recommendations to (1) target STB instead of mental disorders; (2) train counselors to deal directly with STB; and (3) base clinical care on risk stratification and evidence-based, suicide-specific interventions.

The four ATSs to be evaluated in the CAMPUS Trial are as follows:

1. ATS 1: start with 4 to 6 sessions of CAMS. If responding, enter maintenance phase; if not, continue with 1 to 8 sessions of CAMS.
2. ATS 2: start with 4 to 6 sessions of CAMS. If responding, enter maintenance phase; if not, switch to 1 to 8 sessions of CC-DBT.
3. ATS 3: start with 4 to 6 sessions of E-TAU. If responding, enter maintenance phase; if not, switch to 1 to 8 sessions of CAMS.
4. ATS 4: start with 4 to 6 sessions of E-TAU. If responding, enter maintenance phase; if not, switch to 1 to 8 sessions of CC-DBT.

**Table 1:** Assessment battery and schedule of activities for the Comprehensive Adaptive Multisite Prevention of University Student Suicide Trial.

			Screening: week −1		Baseline: week 0		Stage 1													Stage 2																	Follow-up: week 26
							1		2		3		4		5		6		7			8		9		10		11		12		13		14			
**UCC^a^ intake counselor**																																					
	Screening data form^b^	✓																																			
**Student participants**																																					
	Demographic information			✓																																	
	Sexual orientation and gender identity			✓																																	
	CCAPS^c^	✓		✓		✓		✓		✓		✓		✓		✓		✓			✓		✓		✓		✓		✓		✓		✓		✓		
	CGI-S^d^			✓						✓						✓									✓								✓		✓		
	CGI-I^e^									✓						✓									✓								✓		✓		
	AEs^f^ and SAEs^g^			✓		✓		✓		✓		✓		✓		✓		✓			✓		✓		✓		✓		✓		✓		✓		✓		
	DERS^h^			✓												✓									✓								✓		✓		
	DBT-WCCL^i^			✓												✓									✓								✓		✓		
	AAQ^j^			✓												✓									✓								✓		✓		
	SCS^k^			✓						✓						✓									✓								✓		✓		
	Self-efficacy for managing emotions			✓						✓						✓									✓								✓		✓		
	OHS^l^			✓						✓						✓									✓								✓		✓		
	PAI-BOR^m^			✓																																	
	LSC-R^n^			✓																															✓		
	DAST^o^			✓																													✓		✓		
	AUDIT^p^			✓																													✓		✓		
	STCQ^q^					✓												✓																			
	STEQ^r^					✓												✓																			
	CSQ^s^															✓																	✓				
	Academic functioning			✓																													✓		✓		
**Independent evaluators^t^**																																					
	UWRAP^u^			✓						✓						✓									✓								✓		✓		
	CGI-S^v^			✓		✓				✓						✓									✓								✓		✓		
	CGI-I^v^									✓						✓									✓								✓		✓		
	SSI^w^			✓						✓						✓									✓								✓		✓		
	SITBI^x^			✓												✓									✓								✓		✓		
	GAS^y^			✓						✓						✓									✓								✓		✓		
	THI^z^			✓																													✓		✓		
**Research staff**																																					
	Informed consent			✓																																	
	Inclusion and exclusion checklist			✓																																	
	CAMS^aa^ rating scale–3^ab^																																				
	DBT^a^^c^ adherence rating scale^ab^																																				
**Counselor participants**																																					
	Reveal randomization					✓												✓																			
	DIF^ad,ae^			✓																																	
	Treatment compliance item^b^					✓		✓		✓		✓		✓		✓		✓			✓		✓		✓		✓		✓		✓		✓				
	ZSWS^af,ag^	✓																																			
	Exit interview^ah^																																				

^a^UCC: university counseling center.

^b^These measures are exclusively collected in the Titanium electronic medical record and exported at regular intervals throughout the study period.

^c^CCAPS: Counseling Center Assessment of Psychological Symptoms.

^d^CGI-S: Clinical Global Impressions–Severity.

^e^CGI-I: Clinical Global Impressions–Improvement.

^f^AE: adverse event.

^g^SAE: serious adverse event.

^h^DERS: Difficulties in Emotion Regulation Scale.

^i^DBT-WCCL: Dialectical Behavior Therapy Ways of Coping Checklist.

^j^AAQ: Acceptance and Action Questionnaire.

^k^SCS: Suicide Cognitions Scale.

^l^OHS: Optimism Hope Scale.

^m^PAI-BOR: Personality Assessment Inventory–Borderline Features Scale.

^n^LSC-R: Life Stressor Checklist–Revised.

^o^DAST: Drug Abuse Screening Test.

^p^AUDIT: Alcohol Use Disorders Identification Test.

^q^STCQ: Student Treatment Credibility Questionnaire.

^r^STEQ: Student Treatment Expectations Questionnaire.

^s^CSQ: Client Satisfaction Questionnaire.

^t^For all assessments collected as part of an independent evaluator (IE) visit (ie, those assessments not collected specifically at treatment visits), there will be a window of −1 week to +1 week around the scheduled assessment date for the purposes of data collection. IE assessments conducted outside this window will still be collected, and values will be imputed based on when the assessment should have occurred.

^u^UWRAP: University of Washington Risk Assessment Protocol.

^v^These assessments were completed following the students’ last treatment session, which may be earlier than week 6 (for stage 1) or week 14 (for stage 2). The CGI-I and CGI-S were collected at the last treatment sessions at both stage 1 and 2 and at weeks 6 and 14.

^w^SSI: Scale for Suicidal Ideation.

^x^SITBI: Self-Injurious Thoughts and Behaviors Interview.

^y^GAS: Global Assessment Scale.

^z^THI: Treatment History Interview.

^aa^CAMS: Collaborative Assessment and Management of Suicidality.

^ab^Research staff will complete this measure on a random sample of therapy sessions.

^ac^DBT: dialectical behavior therapy.

^ad^DIF: demographic information form.

^ae^Counselor participants will complete this measure once when they enter the study.

^af^ZSWS: Zero Suicide Workforce Survey.

^ag^This measure was also collected at the 6-, 12-, 18-, and 24-month counselor follow-up assessments.

^ah^Counselor participants will complete this interview at the end of their participation in the study.

#### Timeline, Adaptations, and Changes in Design Due to the COVID-19 Pandemic

The CAMPUS Trial was originally scheduled to begin enrollment in May 2020, with all procedures developed for in-person implementation. However, the COVID-19 pandemic necessitated significant changes to the way UCCs provided mental health treatment. Consequently, the CAMPUS Trial procedures and protocols were modified to fit a hybrid tele–mental health (TMH) and in-person format. Training of counselors and assessors occurs online via live Zoom (Zoom Video Communications) sessions. Study treatments and safety monitoring of students are offered both in person and via TMH. Decisions about the type of sessions to hold are made by the counselor based on several factors, including university and UCC policies and location or preference of the student and counselor. Data on treatment modality are collected via electronic medical records.

The results of the CAMPUS feasibility trial showed that a hybrid model, with most sessions being TMH, is feasible, acceptable, and safe when treating college students for suicide risk [27]. Overall, a hybrid model has the greatest future dissemination and implementation potential across UCCs, where the expectation is that treatment delivery will continue to vary within and across settings and will likely retain some combination of in-person and TMH options. After the completion of the feasibility trial, the originally proposed SMART was launched.

### Overview of Study Aims

#### The Primary Aims of the CAMPUS Trial

Aim 1 is to compare the relative effectiveness of the 4 ATSs in reducing university student STB (or both; primary outcomes) and includes the following secondary outcomes: changes in overall distress, depression, anxiety, substance use, eating concerns, academic functioning, health care use, and self-reported ratings of STB.

Aim 2 is to determine whether ATSs beginning with an explicitly suicide-focused intervention (ie, CAMS) produce greater reductions in STB than those beginning with traditional, non–suicide-focused psychotherapies often provided in UCCs (ie, E-TAU).

Aim 3 is to determine whether ATSs providing a comprehensive skills-based, suicide-focused intervention (ie, CC-DBT) to insufficient responders to stage 1 treatments are more effective in reducing STB than a less intensive suicide-focused approach (ie, CAMS).

#### The Secondary Aims of the CAMPUS Trial

Aim 4 is to evaluate treatment-specific mediators of change, including changes in suicide-focused processes, emotion regulation, and self-efficacy.

Aim 5 is to evaluate nonspecific predictors and moderators of treatment response, including number of lifetime SAs [28], features of borderline personality disorder (BPD) [29], baseline distress [30], sexual orientation and gender minority self-identification [31,32], and comorbid substance use [33].

#### The Exploratory Aims of the CAMPUS Trial

Aim 6 is to assess the implementation outcomes outlined by Proctor et al [34] using the Quality Implementation Framework [35].

Aim 7 is to explore counselor experiences working with university students presenting with SI.

Aim 8 is to determine whether CAMS is associated with greater reductions in STB than E-TAU within stage 1 and whether CC-DBT is associated with greater reductions in STB than continued CAMS within stage 2.

## Methods

### Participants

In total, 2 study populations will participate in the CAMPUS Trial: university students and UCC counselors. A target sample size of 480 university students seeking treatment at their counseling center will participate in the CAMPUS Trial from 4 universities: Duke University; Rutgers University–Newark; University of Nevada, Reno; and University of Oregon. To participate in the study, students must (1) be officially enrolled at the university; (2) be eligible to receive counseling services; (3) be seeking treatment services at their counseling center; (4) have not received treatment services at their counseling center within the previous 3 months; (5) be aged between 18 and 25 years; (6) endorse moderate to severe SI on the Counseling Center Assessment of Psychological Symptoms [36], other similar standard questionnaires, or via self-report to the clinician during intake; (7) present with SI considered severe enough to be a focus of treatment as determined by the intake counselor; and (8) agree to video or audio recording of all therapy and assessment sessions. A target sample size of 20 counselors will be recruited for participation across sites. Inclusion criteria for counselor participants are (1) current employment as a counselor or trainee at the counseling center; (2) agreement to participate for at least 1 year; (3) endorsement of a willingness to work with suicidal college students, attend all CAMS and CC-DBT trainings, and participate in weekly consultation groups; and (4) completion of measures about themselves and the students they treat as part of the study.

Student participants will be recruited through standard intake procedures at each UCC site from August and September 2022 to April and May 2024. As part of these procedures, all students seeking services are assessed for SI. Students who meet study eligibility criteria are offered the opportunity to meet with a member of the research team to learn more about the study and complete the informed consent process.

Counselor participants are initially identified by the UCC directors or hired as trainees to work at the UCC and be part of the study. All interested counselors are referred to the site investigators for possible study participation and to complete the informed consent process.

### Ethical Considerations

To streamline and expedite the institutional review board (IRB) review process across the multiple participating institutions and to ensure consistency in ethical review, this study uses a single IRB, specifically, the Duke University Health System IRB. Written informed consent is obtained for all participants and captured via the REDCap (Research Electronic Data Capture; Vanderbilt University) eConsent framework. Each site IRB and the Duke single IRB approved the consent forms and protocol before study initiation (approval: Pro00104815).

All research data will be kept in the REDCap database, which is managed by the Duke data center. REDCap data are encrypted both at rest and in transit. The Duke University Medical Center database-hosting infrastructure was audited by the Duke Information Security Office for compliance with HIPAA (Health Insurance Portability and Accountability Act) and Duke Health data security policies. With the participants’ approval, deidentified data from this study will also be submitted to the NIMH Data Archive (NDA) at the National Institutes of Health (NIH) and stored indefinitely. Digital-based data will not be submitted to the NDA at the NIH. During the course of the study, an individual participant can choose to withdraw consent to have their data stored at the NIH. Student participants are reimbursed for completing each assessment based on the following rates: US $40 at baseline, US $20 at week 3, US $40 at week 6, US $20 at week 10, US $50 at week 14, and US $50 at week 26. The total reimbursement for any student who completes all assessments is US $220.

### Design Overview

**Figure 1:**
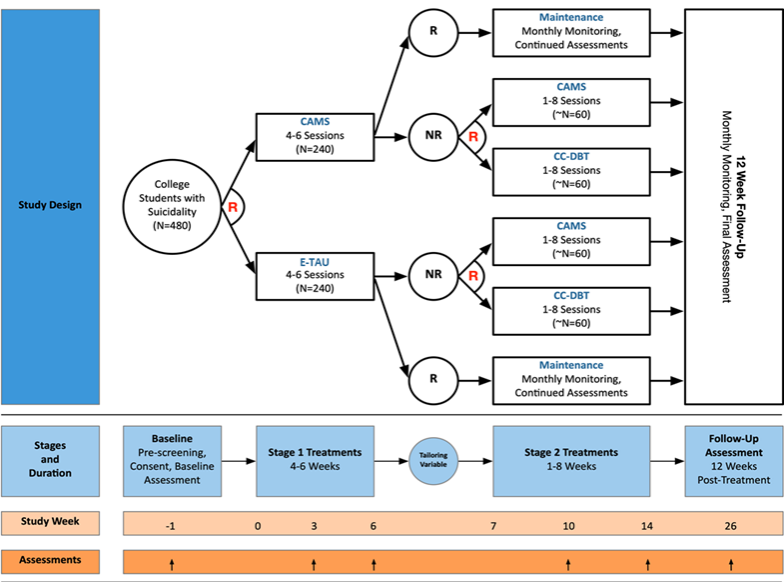
Comprehensive Adaptive Multisite Prevention of University Student Suicide Trial study design. CAMS: Collaborative Assessment and Management of Suicide; CC-DBT: counseling center dialectic behavior therapy; E-TAU: enhanced treatment as usual; NR: nonresponder to treatment; R (black): responder to treatment; R (red): randomization point.

As illustrated in Figure 1, the CAMPUS Trial used a 2-stage SMART design. In stage 1, all student participants who consent and meet study inclusion criteria are randomized to 4 to 6 weeks of either CAMS or E-TAU. Sessions are individual and scheduled weekly. Starting at the end of session 4 (noting that all students are offered at least 4 therapy sessions), and after each subsequent session, counselors use their ratings of student improvement in STB since baseline to inform treatment decisions. Between sessions 4 and 6, students whose suicidal risk improves above a specified threshold (refer to the Tailoring Variable section) are defined as treatment responders and move into the maintenance phase of the study, which involves monthly contacts via email by research staff. Students whose suicide risk has not improved sufficiently by session 6 are classified as treatment nonresponders and rerandomized to an additional 1 to 8 weeks of 1 of 2 stage 2 treatments: CAMS or CC-DBT.

Assessments are administered by independent evaluators (IEs) blind to treatment condition at week 0 (baseline), week 3 (mid–stage 1), week 6 (end of stage 1), week 10 (mid–stage 2), week 14 (end of stage 2), and week 26 (3-month follow-up).

### Randomization

To maintain reasonably good balance among the 4 ATSs, student participants are randomized using a stratified block randomization procedure implemented via the REDCap randomization module [37,38]. Stratification variables used in this procedure are treatment site (Duke, Rutgers, University of Nevada, and University of Oregon), self-identified student sex at birth (male or female), history of previous SAs (yes or no), and current use of psychotropic medications (yes or no). The site principal investigator (PI) or delegate signs off on each randomization.

### Sample Size and Power Estimates

Statistical power was calculated for aims 1 to 5 using a continuous outcome. Estimated effect sizes for moderation and mediation aims based on the sample size required for aim 1 are also reported. Statistical power for exploratory aims was not calculated. Power is dependent on the respective treatment comparisons of interest as well as attrition and response rates per stage. To represent the respective contrasts of interest, the ATSs embedded by design are listed in Table 2. All sample size and power estimates were calculated based on the target sample size of 480.

The primary goal of aim 1 is to identify the most effective ATS among the 4 embedded strategies (A+B, A+C, D+E, or D+F, as shown in Table 2) that leads to the greatest reduction in STB. This aim focuses on finding the best-performing ATS rather than testing a hypothesis. The sample size of 480 was found to ensure an 80% probability of correctly identifying the ATS with the lowest mean outcome, assuming that such a strategy exists. This calculation is based on the method outlined by Scott et al [39] and Oetting and Levy [40] and assumes equal marginal variance of outcomes across the ATSs, a normally distributed mean outcome, a type-I error rate of 2.5% where we adjust the standard 0.05 level by a factor of 2 for the 2 primary outcomes of STB, a standardized effect size of 0.50, and no more than 50% attrition by stage 2.

The objective of aim 2 is to examine the main effect of the stage 1 treatments—whether it is more effective to begin an ATS with a suicide-focused intervention (CAMS) or with traditional counseling (E-TAU). This comparison allows for the evaluation of the main effect of the 2 initial treatments averaged across the second-stage treatments among stage 1 nonresponders (A+B+C vs D+E+F). The test statistic for this comparison is the standard test for a 2-group comparison in large samples and is not specific to the SMART design. A 2-sided, independent-sample *t* test with a type-I error rate of 2.5% will provide 85% power to detect a small effect size (*d*=0.30) on the primary outcome, with a target sample of 480 participants randomized to the 2 stage 1 treatments. Assuming an SD of 7.0 on the Scale for Suicidal Ideation (SSI) [41] (based on previous studies, including pilot data), this effect size corresponds to a clinically meaningful difference of 2 points on the SSI.

The purpose of aim 3 is to evaluate the main effects of stage 2 treatments—specifically, whether CC-DBT is more effective than CAMS among those who do not respond to stage 1 treatments (B+E vs C+F). Assuming a 50% response rate to stage 1 treatments (n=240) and a 20% attrition rate, approximately 48 participants will be eligible to be randomized to each of the 4 stage 2 treatment conditions (B, C, E, and F). On the basis of the method outlined by Oetting and Levy [40], and assuming a within-person correlation of 0.60 with a type-I error rate of 2.5%, we will have 80% power to detect a small to moderate effect size (*d*=0.34) on the primary outcome between the CC-DBT (n=96) and CAMS (n=96) stage 2 treatments.

Best practices for power calculations in mediation and moderation models within SMART designs are still a topic of debate. For mediation, we will follow the work by Fritz and MacKinnon [42], who report sample size requirements to ensure 80% power under the sequential regression framework, and the formulas by Vittinghoff et al [43]. Assuming a medium effect size for the intervention on the mediator and the outcome (covarying the mediator) and a type-I error rate of 2.5%, a total sample size of at least 166 is required. Therefore, our design, with a total sample size of 96 in each of the 2 stage 2 treatments (or n=192), is sufficiently powered to detect medium-sized mediation effects within stage 2. For moderation, based on the power tables by Cohen, our sample size of 480 at the start of stage 1 and at least 96 participants per treatment in stage 2 are more than sufficient to detect a medium to large effect size for a moderator.

**Table 2:** The 4 adaptive treatment strategies embedded in the implemented sequential multiple-assignment randomized trial.

Adaptive treatment strategy, stage 1 treatment, and status at the end of stage 1		Stage 2 treatment	Strategy denomination^a^	Cells involved in comparisons	
**1—CAMS^b^**					A+B
	Responder	Maintenance and monitoring	A		
	Insufficient responder	Continue CAMS	B		
**2—CAMS**					A+C
	Responder	Maintenance and monitoring	A		
	Insufficient responder	Switch to CC-DBT^c^	C		
**3—E-TAU^d^**					D+E
	Responder	Maintenance and monitoring	D		
	Insufficient responder	Switch to CAMS	E		
**4—E-TAU**					D+F
	Responder	Maintenance and monitoring	D		
	Insufficient responder	Switch to CC-DBT	F		

^a^For simplicity, the letters A to F are used in this column to denote the corresponding stage 2 treatment listed in the previous column.

^b^CAMS: Collaborative Assessment and Management of Suicidality.

^c^CC-DBT: counseling center dialectical behavior therapy.

^d^E-TAU: enhanced treatment as usual.

### CAMPUS Trial Treatments

#### Overview

In developing ATSs for university students struggling with thoughts of suicide, we were interested in addressing 2 specific questions that often arise when managing this population. First, should STB be the focus of care ahead of other concerns raised by the students, or should the counselor monitor STB but provide more general supportive care? This question is addressed by comparing outcomes between students who started treatment with E-TAU versus those who started with CAMS. Second, for those students who are insufficient responders to their initial course of care, should the counselor stay the course by offering more sessions, or should the counselor switch to a new treatment approach? This question is addressed by our treatment sequences, where nonresponders either continue the same treatment across stages or switch treatments. The treatment sequences also allow us to address whether it is best to use a more intensive skills-based, suicide-focused approach (CC-DBT) or a more streamlined weekly approach (CAMS) for the second line of treatment.

#### E-TAU Intervention

Students randomly assigned to E-TAU receive the customary individual therapy that they would receive at that UCC, which can also be supplemented with group participation or medication referral at the counselor’s discretion. In E-TAU, counselors are allowed to determine the level of care and therapeutic modality depending on the needs of the participant. For clinical consultation, counselors follow their clinic’s procedures (eg, consulting with the clinical director and attending case management meetings). The only stipulations provided to counselors offering E-TAU is that sessions should be weekly even if this is not the norm at their UCC, and they are required to monitor student participants’ STB to rate their response to treatment. Accordingly, this treatment is designed as *enhanced* treatment as usual (TAU). Counselors conducting E-TAU treatment are asked to provide counseling as usual for a period of 4 to 6 weeks but are asked not to use CAMS (eg, suicide status forms and sitting next to the client) or CC-DBT (eg, diary card and DBT skills)–specific strategies. Counselors are informed that all their sessions are recorded and some sessions will be randomly rated on adherence to ensure that no CAMS and CC-DBT strategies are used.

#### CAMS Intervention

CAMS is an evidence-based, suicide-focused framework that was first developed and studied in a UCC [44]. CAMS is a problem-focused treatment that targets client-defined suicidal *drivers* or issues that lead to SI [21]. To date, 11 open clinical trials of CAMS have reliably shown significant reductions in SI and overall distress [45,46], 3 of these with university students [47-49]. Furthermore, 7 randomized controlled trials (RCTs) have found that CAMS reduced SI compared to control care as usual [50], led to reductions in overall distress while increasing hope and patient satisfaction in comparison to control care [51], performed similarly to DBT in terms of reductions in nonsuicidal self-injury (NSSI) and SAs [52], lowered risk of SA following discharge from an inpatient setting compared to TAU [53], and reduced SI and depression more than TAU in a university student population [49]. A 9-study meta-analysis showed that CAMS significantly reduced SI and overall symptom distress, increased hope and decreased hopelessness, and increased retention to care (when compared to control care) [54]. Finally, CAMS can be used effectively via telehealth [55] and has been shown to be cost-effective [56].

UCCs need an efficient and effective stepped-care approach to treating students with STB, delivering more intensive treatments only to those who need them. CAMS is an ideal first-line intervention because it is flexible and easy to train for, and several UCCs report already using CAMS [14]. While there are other empirically based suicide-focused approaches [57,58], they either have not been tested within a UCC or require more extensive training than CAMS.

#### CC-DBT Intervention

DBT is an empirically validated treatment for complex clinical presentations, including BPD, SI, and NSSI. Comprehensive DBT (which includes individual therapy, skills group, between-session skills coaching, and peer consultation for counselors) has been noted to result in gains for clients across a variety of domains, including SI, BPD, SAs, NSSI, hospitalizations, and social functioning [59,60]. DBT is based on a skills deficit model that suggests that BPD is a disorder of emotion dysregulation stemming from deficits in interpersonal, emotion regulation, and distress tolerance skills. STB are viewed as maladaptive problem-solving behaviors reinforced by an immediate reduction in emotional arousal or by the environment’s response [61]. Thus, DBT focuses on teaching skills from 4 domains (mindfulness, interpersonal effectiveness, emotion regulation, and distress tolerance) and facilitating the replacement of maladaptive behaviors with skillful behaviors.

Members of the CAMPUS Trial research team previously conducted the only RCT to date using comprehensive DBT for university students with STB [23]. Compared to an optimized control condition, 7 to 12 months of DBT led to significantly greater decreases in SI, depression, NSSI events, and BPD symptoms and greater improvements in social adjustment. DBT was particularly effective for students who were suicidal and were lower functioning at baseline. However, some students dropped out before the end of treatment due to improvement, suggesting that a less intensive and shorter approach might be adequate for certain students. Although there has only been 1 RCT with DBT at UCCs, open trials have also been conducted with DBT at UCCs [62,63], and more than a dozen studies have investigated the use of DBT skills groups in UCCs [24]. A recent survey concluded that approximately one-third of UCCs already use some elements of DBT [24], and a significant body of research indicates that DBT is effective for the types of clinical presentations at UCCs [64].

The CAMPUS Trial evaluates an adaptation of comprehensive DBT for UCCs (CC-DBT) as a second-stage treatment for students who were insufficient responders to CAMS or E-TAU in stage 1. Our adapted form of DBT (CC-DBT) was designed for dissemination within UCCs, with adaptations based on feedback and data on treatment adherence from the CAMPUS feasibility trial [27]. The modifications prioritized key elements of DBT (diary card self-monitoring, chain analyses, skills teaching, and generalization) to facilitate ease of use and fit a timeline more consistent with UCC practices and client preference. The skills training portion was reduced to 6 sessions teaching a core subset of the DBT skills package. CC-DBT also included weekly peer consultation meetings for the counselors that took place remotely and across sites, and depending on site policies, phone skills coaching was offered during business hours.

#### Maintenance Phase

Student participants who are classified as responders during either stage 1 or stage 2 enter the maintenance phase. Students are able to continue receiving UCC services during this phase but do not receive any additional CAMPUS Trial treatment. Students continue to complete regularly scheduled IE assessments and remain in the maintenance phase until the end of their study participation at 26 weeks. During this phase, the study team contacts students every 4 weeks via email to provide mental health and crisis resources. The 4-week contact schedule continues until their final assessment point. Relapses in STB during the maintenance phase can be reported during the scheduled IE assessments, in response to a 4-week contact, or by the student spontaneously contacting a member of the CAMPUS Trial clinical or research team. In these instances, students are referred to their UCC for standard care. Treatments received outside of the study are documented via the Treatment History Interview [65] and a review of the electronic medical record at the UCCs.

### Assessments

#### Overview

The CAMPUS Trial assessment battery (summarized in Table 1) uses multiple informants to capture comprehensive information about aspects of student functioning, treatment experiences, and program implementation. The informants include (1) student participants; (2) IEs who conduct semistructured student assessments; and (3) counselor participants who provide study treatment, assess student response to treatment, and provide information about their own study experiences. Measures were adopted if they (1) had adequate psychometric characteristics, (2) were used in previous suicide treatment studies, (3) were shown to be sensitive to change, (4) had been used in previous studies with university students, (5) mapped onto the NIMH’s Research Domain Criteria categories (Negative Valence Systems, Positive Valence Systems, Cognitive Systems, Social Processes, and Arousal and Regulatory Systems) [66,67], (6) were directly relevant to the application of CAMS or DBT, and (7) allowed for reduced burden to student participants. Assessment manuals for IEs and counselor participants fully specified the rationale and procedures for each of the assessment measures. All self- and IE-reported assessments are collected from student participants regardless of treatment status.

#### The Primary Outcome Measures

The primary outcome is reduction in STB as measured by the IEs at each assessment using 2 standardized interviews: the SSI [41] and the Self-Injurious Thoughts and Behaviors Interview (SITBI) [68]. Changes in SI are assessed using SSI total scores. Suicidal behaviors, including NSSI and SAs, are captured via the SITBI. The SSI interview is presented in a semistructured format and consists of 19 items that include 3 subscales of SI: passive suicidal desire, active suicidal desire, and specific plans for suicide. Each item is rated on a 3-point Likert-type scale from 0 to 2 and then summed, with higher total and subscale scores indicating greater severity of SI. The SITBI is also presented in a semistructured format and comprises modules that assess SI, suicide plans, suicide gestures, SAs, and NSSI. The IEs are trained and certified on both interview measures as described in the following sections. Recognizing that measurement of suicide-related behaviors is fraught with challenges [69], a separate study will also explore defining suicide risk as a composite measure of SI, SAs, and NSSI.

#### Tailoring Variable

In the context of SMART designs, tailoring variables are characteristics of participants that trigger adaptations to the ATSs. The tailoring variable used in this study is a modified version of the Rating of Clinical Improvement–Suicidality (RCI-S) scale, an adapted version of the Clinical Global Impressions scale [70] designed to assess overall improvement in suicide risk. Beginning in session 4, and following each subsequent session, counselors use the 4-point Likert-type RCI-S to rate the students’ treatment response trajectory with respect to their suicidal symptoms since baseline from *Responder* (1) to *Significant Worsening* (4). Students who are rated as responders move into the maintenance phase of the trial. Insufficient responders (those with RCI-S ratings of ≥2) at the end of stage 1 are eligible to be rerandomized into 1 of the 2 stage 2 treatments.

#### Secondary Measures

Secondary measures (also listed in Table 1) were designed to capture overall distress, depression, social and generalized anxiety, substance use, eating concerns, academic functioning, and clinical global impressions by both participants and assessors of severity and improvement in suicidal risk. These measures are included to allow for the examination of mediators, predictors, and moderators of treatment response, which could be incorporated as secondary tailoring variables in later studies or during dissemination. Quantitative and qualitative assessments to monitor implementation activities and address barriers that arose during the study are also collected. The implementation monitoring plan was guided by three components: (1) critical steps of the Quality Implementation Framework [35], (2) process evaluation and implementation monitoring outlined by Saunders [71], and (3) implementation outcomes specified by Proctor et al [34]. These measures include student and counselor views on treatment experiences and satisfaction as well as comparisons between in-person counseling and TMH formats.

### Student Safety Procedures

#### Overview

Given the inherent risks of the population under study, particular care was taken to develop and implement strategies to ensure participant safety. These include procedures for adverse event (AE) monitoring, detailed manual-based protocols to minimize risk of worsening symptoms and manage emergent crises, and procedures to quickly detect and alert research staff when an AE occurs. The study protocol allows for an increase in session frequency if needed, and student participants who worsen significantly might be removed from the study and referred to a higher level of care if needed. The CAMPUS Trial is also monitored by a blinded NIMH Data Safety and Monitoring Board (NIMH-DSMB). The NIMH-DSMB meets 3 times per year to review and approve all protocol changes, enrollment data, safety data, and data integrity.

AEs and serious AEs (SAEs) are monitored at each treatment and assessment visit. For the purposes of this study, the following events are considered AEs: (1) breach of confidentiality, (2) evidence of coercion to participate, (3) evidence of distress during assessments (using data from the University of Washington Risk Assessment Protocol) [72], and (4) significant increase in SI (relative to baseline) as rated by counselor participants (at each treatment visit) and IEs (at each assessment visit). SAEs include (1) suicide death, (2) nonsuicide death, (3) SA (not death) with nonzero intent to die, (4) inpatient hospitalization, and (5) emergency department visit.

During treatment visits, counselor participants document any new AEs or SAEs spontaneously reported during therapy sessions. Unsolicited events that meet the definition for AEs or SAEs prompt further inquiry by the research team to ascertain onset; severity; relatedness to treatment; outcome; and measures taken to address the event, if any. During each assessment visit, AEs and SAEs are also assessed and monitored through general inquiry by the IEs. IEs also implement the University of Washington Risk Assessment Protocol [72] at each assessment to monitor suicide risk more closely.

#### Special Considerations for Risk Management During Remote Assessments and Treatment Visits

Because of the remote nature of TMH care, each student’s current location, as well as emergency contact numbers for third-party involvement (eg, parents or roommates approved by the student), are obtained should there be a worsening of symptoms. IEs are also trained to gather information for secondary backup methods, even tertiary when possible, for reconnecting with students in case of technological issues or an unstable internet connection. Before initiating any remote assessments with student participants, IEs receive basic training in TMH by viewing the first 3 segments of the American Psychological Association Telepsychology Best Practices 101 series webinars [73].

### Quality Assurance

#### Overview

UCC counselors wishing to be study counselors undergo a rigorous certification process for CAMS and CC-DBT. For CAMS, certification requirements occur in stages. Before treating a student participant, counselors must (1) read all CAMS treatment-related materials, including the study protocol; (2) watch a 3-hour CAMS online training course; and (3) attend a full-day CAMS role-play workshop. Following these trainings, counselors must (1) attend weekly cross-site consultation calls and (2) receive a minimum of *good* on CAMS adherence ratings for 3 sessions or until the student completed treatment (ie, if suicide risk resolved in <6 sessions), with individual feedback to clinicians provided via email. Once counselors are certified as study adherent to CAMS, 2 CAMS sessions from each student participant will be randomly selected and rated for adherence to the CAMS model.

Before treating a student participant using CC-DBT, counselors must (1) read all CC-DBT treatment-related materials, (2) attend a 4-day online DBT training workshop and a 1-day online skills training workshop, and (3) attend weekly cross-site consultation team meetings following the trainings. Recordings of each session of a counselor’s first CC-DBT student are reviewed for adherence by DBT expert clinicians, and feedback is provided via email. CC-DBT sessions are rated for adherence until each counselor demonstrates that they can meet the threshold for adherence on the DBT Adherence Checklist for Individual Therapy (a score of ≥22) [74] on at least 2 sessions, with at least one of these sessions being a later session of treatment (ie, sessions 3-8). Once counselors are certified as study adherent to DBT, 2 CC-DBT sessions from each student participant will be randomly selected and rated for adherence to the DBT model.

IEs also undergo a rigorous certification process. IEs must (1) read all study-related materials, including the study protocol; (2) attend a 2-day online training covering each of the IE-administered outcomes; (3) complete *on your own* practice administering study interviews and outcomes; (4) watch and score a standardized video of an assessment; (5) practice administering an assessment with a standardized practice participant; and (6) attend twice monthly cross-site assessment supervision meetings where scoring questions and concerns are discussed (note: SSI and SITBI consultant Dr Julia Harris will also be present to facilitate challenging classification of questions and concerns).

#### Data Management

All research data are kept in an encrypted REDCap database managed by the Duke data center at the Duke University Medical Center. With participants’ approval, deidentified data will also be submitted to the NDA. Study procedures include detailed instructions for data acquisition, processing, and upload to the REDCap platform. Any data collected on paper forms are double entered by site study staff and directly uploaded into the REDCap database using specific electronic case report forms. Many data validation rules (eg, out-of-range values and skip patterns) are enforced by the electronic data capture system during data entry. The Duke data center continually monitors data quality throughout the study, conducting daily checks using the REDCap auditing system to annotate and document any inconsistencies or possible errors to be resolved by site study staff.

### Design Challenges and Alternatives Considered

In the process of refining the CAMPUS Trial design, we considered the following design alternatives but ultimately did not use them for ethical, pragmatic, and clinical reasons.

#### Using Different Counselors for Different Treatments

The CAMPUS Trial team carefully considered the option of using different counselors to implement the different treatments. Although this is desirable from an efficacy-based perspective, we discarded this design option due to compelling ethical and clinical, pragmatic, and methodological reasons and decided to have the same counselors deliver all interventions. Requiring student participants with unresolved STB (or both; who might struggle with fears of abandonment) to switch counselors at the end of stage 1 could lead to an escalation in STB [29] or increased likelihood of treatment dropout. Previous research has demonstrated that keeping the same counselor across treatment transitions in a UCC is associated with half as many treatment dropouts and fewer sessions overall [75]. Furthermore, this design better reflects what would happen in the *real world* in that counselors often evolve or change their treatment plans if a client is not responding rather than refer them to another counselor. Quality assurance procedures were developed to assess for contamination of CAMS or CC-DBT into E-TAU.

#### Mandating a Full Course of Treatment

A priority of the CAMPUS Trial is to develop evidence-based ATSs that are feasible to implement in UCCs, which must operate with limited resources and in the context of an academic calendar. Mandating a full course of treatment for all students is impractical in the UCC setting. Provided that students receive a *minimum dose* of 4 treatment sessions, they are not required to continue study treatment once they are classified as treatment responders. In other words, students only receive as much treatment as they need to improve. We also allow for flexibility in the delivery of treatments to accommodate the academic calendar*.* All treatment sessions are conducted either in person or remotely via a HIPAA-compliant TMH platform. Thus, continuity of treatment during short breaks is facilitated by the use of a telehealth option. For longer academic breaks (eg, winter break), students are permitted to take a *break* from CAMPUS Trial treatment while they are on a scheduled break (and often unable or ineligible to receive UCC services due to residing out of state) and then continue where they left off upon their return to campus. This is an essential adaptation to the UCC setting and was considered in terms of recruitment rates. Breaks will also be considered in the analyses (eg, dosage defined as number of sessions and not time since randomization per stage and number of additional treatment sessions received).

#### Deviations From the Original Protocol

The protocol presented in this publication differs from the initial design that was peer reviewed by the funding agency. These modifications were necessary due to the unforeseen impact of the COVID-19 pandemic, which posed challenges that could not have been anticipated at the time of initial planning. To ensure participant safety and adapt to logistical constraints, we implemented substantial changes in the trial design and methodology. These modifications were carefully considered and implemented in consultation with the scientific advisory board, UCC implementation advisory board, and program officer from the funding agency. Each of these stakeholders provided invaluable guidance to ensure that the changes maintained the scientific integrity of the study while allowing for adaptation to the evolving situation. Any adjustments to the initial design were transparently documented, reviewed, and approved by the funding agency, each site’s IRB, and the assigned NIMH-DSMB. This approach aimed to ensure that the study findings remain as unbiased as possible despite the external constraints encountered.

Most of the deviations from the original protocol were necessitated by the COVID-19 pandemic and included procedures to accommodate UCC TMH services, including remote treatment, assessments, and safety monitoring. Counselor trainings were also modified to accommodate remote delivery. However, additional modifications arose from lessons learned during the subsequent feasibility study required by the NIMH and NIMH-DSMB. These included changes to the assessment battery to reduce participant burden and improve remote data collection procedures and adaptations to comprehensive DBT to better fit the UCC setting and academic calendar.

### Organization and Study Management

To ensure scientific integration of research procedures, overall managerial and administrative responsibilities rest with the CAMPUS Trial steering committee (SC), which comprises the PIs and coinvestigators from each site, principal statistician, and NIMH-DSMB liaison. As relevant, additional team members, including project coordinators, participate in SC meetings. The SC is responsible for all decisions concerning the overall research program, including plans for data analysis and publications. The SC holds weekly videoconference calls to monitor the overall course of the study, including recruitment, retention, and any out-of-protocol deviations. In case of disagreements, each site has 1 vote, and the statistician will break a tie. Various subcommittees (eg, quality assurance, treatment, and assessment) will be formed across investigators and consultants, and these subcommittees present potential challenges and solutions to the SC (these actions are documented via minutes).

There are also two advisory boards: (1) a scientific advisory board composed of a suicide researcher with expertise in multisite trials, a psychiatrist, a research scientist with expertise in SMART methodology, and a UCC expert; and (2) a UCC implementation advisory board composed of the 4 site UCC directors or clinical directors. The PIs, coinvestigators, and project coordinators attend an annual in-person study meeting and also meet at professional conferences throughout the study period.

## Results

Student participant recruitment began on October 25, 2022, and ended on May 16, 2024. Data collection was complete in November 2024, and as of April 2025, data analysis is underway. Full results will be available in 2025.

## Discussion

### Expected Findings

The CAMPUS Trial offers a model for future SMART designs in UCCs and similar settings. Primary and secondary results of the CAMPUS Trial will offer important clinical decision-making guidelines for informing the treatment of university students presenting with STB in UCCs. A previous pilot SMART [20] laid the groundwork for the CAMPUS Trial, which was designed to improve upon previous limitations by increasing the sample size, incorporating multiple UCCs, integrating remote assessments and treatments, and evaluating ATSs with shorter durations to enhance feasibility and broader adoption across diverse UCC settings. We hypothesized that starting with CAMS and then (1) ending treatment and entering maintenance if student participants improve (stage 1) or (2) switching to a more intensive treatment if there is insufficient improvement (stage 2) would be the most effective treatment sequence for students who are suicidal seeking services at UCCs.

The hybrid intervention format of the CAMPUS Trial represents an important contribution to efforts to improve treatment of suicidality via telehealth. Before the COVID-19 pandemic, the prevailing belief was that clients with STB posed too great a risk for treatment via TMH. The CAMPUS Trial feasibility pilot study demonstrated that TMH is a feasible and acceptable modality for delivering suicide-focused treatments [27]. Results from the trial may provide additional insights into whether treatment modality impacts response rates and provide guidance to UCCs and other organizations on how to deliver suicide-focused treatments.

### Limitations

First, a key concern is the heterogeneity of E-TAU. Because E-TAU was not standardized across sites, variations in the type and theoretical orientation of care received by student participants have the potential to influence outcomes. While this variability reflects real-world clinical practice, it can present challenges for interpretation. To address this, we implemented systematic monitoring of E-TAU, including quality control ratings to assess for contamination of CAMS and CC-DBT principles into E-TAU, and gathered information on clinicians’ theoretical orientations and treatment choices when delivering E-TAU. However, another important limitation is that E-TAU may not fully reflect true TAU at UCCs. The CAMPUS Trial counselors were trained in suicide-focused treatments and served as their own controls, which may have influenced the way in which they delivered TAU even in the absence of direct contamination from CAMS and CC-DBT. Although quality control measures were implemented to prevent cross-treatment contamination, the fact that all counselors had formal training in structured suicide-focused approaches means that the TAU they provided may be different from what is typically offered in UCCs. This could limit the generalizability of findings regarding the effectiveness of standard TAU provided outside the trial. Although this design introduces some challenges, treatment provision at UCCs varies considerably, and this reflects the reality of the setting. Furthermore, future secondary studies offer the potential to make a significant contribution to the field of suicide prevention by characterizing TAU in the context of suicidal risk and further examining how clinician training influences TAU delivery.

Because the CAMPUS Trial participants are university students, there can be concern about the lack of generalizability of the results to broader populations. However, the findings of this study should have relevance beyond university settings. Young adults in higher education represent a diverse group with varying backgrounds, mental health histories, and treatment needs, making them an important population for studying suicide-focused interventions. In addition, many of the challenges addressed in the CAMPUS Trial—such as accessibility of care, personalizing the provision of care to improve outcomes, and the integration of TMH—are applicable across different clinical settings. The CAMPUS Trial results can inform best practices for delivering suicide-focused treatments in community mental health centers, primary care settings, and other outpatient environments. Furthermore, the use of ATSs to tailor interventions based on individual response patterns provides a flexible framework that can be adapted for different populations, including those in nonuniversity settings.

## Appendix

Multimedia Appendix 1 Peer review reports from the ZMH1 ERB-K (04) - National Institute of Mental Health Special Emphasis Panel (National Institutes of Health, USA).

Multimedia Appendix 2 Authors’ response to peer reviews from the ZMH1 ERB-K (04) - National Institute of Mental Health Special Emphasis Panel (National Institutes of Health, USA).

Multimedia Appendix 3 Author commentary on original peer reviews.

## Abbreviations

AE: adverse event
ATS: adaptive treatment strategy
BPD: borderline personality disorder
CAMPUS: Comprehensive Adaptive Multisite Prevention of University Student Suicide
CAMS: Collaborative Assessment and Management of Suicidality
CC-DBT: counseling center dialectical behavior therapy
DBT: dialectical behavior therapy
E-TAU: enhanced treatment as usual
HIPAA: Health Insurance Portability and Accountability Act
IE: independent evaluator
IRB: institutional review board
NDA: National Institute of Mental Health Data Archive
NIH: National Institutes of Health
NIMH: National Institute of Mental Health
NIMH-DSMB: National Institute of Mental Health Data Safety and Monitoring Board
NSSI: nonsuicidal self-injury
PI: principal investigator
RCI-S: Rating of Clinical Improvement–Suicidality
RCT: randomized controlled trial
REDCap: Research Electronic Data Capture
SA: suicide attempt
SAE: serious adverse event
SC: steering committee
SI: suicidal ideation
SITBI: Self-Injurious Thoughts and Behaviors Interview
SMART: sequential multiple-assignment randomized trial
SSI: Scale for Suicidal Ideation
STB: suicidal thoughts or behaviors
TAU: treatment as usual
TMH: tele–mental health
UCC: university counseling center

## Data Availability

The datasets generated or analyzed during this study are available in the National Institute of Mental Health Data Archive.
