# Supplementary material for: The Comprehensive Adaptive Multisite Prevention of University Student Suicide Trial: Protocol for a Randomized Controlled Trial
Source: JMIR Res Protoc. 2025 Apr 22;14:e68441. doi: 10.2196/68441 (PMC12056417; doi:10.2196/68441)
Supplement: Multimedia Appendix 1 [file resprot_v14i1e68441_app1.pdf]

SUMMARY STATEMENT

PROGRAM CONTACT:
MARY Rooney
301-827-1325
mary.rooney@nih.gov

(Privileged Communication)

Release Date: **04/15/2019**
Revised Date:

Application Number: [redacted]

Principal Investigator

[redacted]

Applicant Organization: [redacted]

Review Group: **ZMH1 ERB-K (04)**
National Institute of Mental Health Special Emphasis Panel
NIMH Clinical Trials Peer Review Meeting

Meeting Date: **04/08/2019**
Council: **MAY 2019**
Requested Start: **07/01/2019**

RFA/PA: **MH18-700**
PCC: **85-TPSP**

Project Title: **1/4 Adapting Treatments for Suicidal College Students: A Multisite Trial**

SRG Action: **Impact Score:28**
Next Steps: **Visit https://grants.nih.gov/grants/next_steps.htm**
Human Subjects: **48-At time of award, restrictions will apply**
Animal Subjects: **10-No live vertebrate animals involved for competing appl.**
Gender: **1A-Both genders, scientifically acceptable**
Minority: **1A-Minorities and non-minorities, scientifically acceptable**
Age: **3A-No children included, scientifically acceptable**

Project Year	Direct Costs Requested	Estimated Total Cost
1	[redacted]	[redacted]
2		
3		
4		
TOTAL	[redacted]	[redacted]

ADMINISTRATIVE BUDGET NOTE: The budget shown is the requested budget and has not been adjusted to reflect any recommendations made by reviewers. If an award is planned, the costs will be calculated by Institute grants management staff based on the recommendations outlined below in the COMMITTEE BUDGET RECOMMENDATIONS section.

[redacted]

PROTECTION OF HUMAN SUBJECTS: UNACCEPTABLE

RESUME AND SUMMARY OF DISCUSSION: Submitted in response to RFA-MH-18-700, this Collaborative R01 resubmission proposes a 4-site study to test evidence-based adaptive treatment strategies (ATS) to address suicidal risk in college students seeking treatment at a college counseling center which are frequently overburdened. The study would use a two-stage, sequential, multiple assignment, randomized trial (SMART) with 700 students randomized to Collaborative Assessment and Management of Suicidality (CAMS) or Treatment as Usual (TAU). Non-responders will be re-randomized to one of two second-stage higher intensity/dosage intervention options: CAMS (continued or administered for the first time) or Comprehensive Dialectical Behavior Therapy (DBT). The public health significance of the project is high, given the increasing rates of suicide and suicidal ideation among college students. Also, the well-crafted SMART design will inform clinical decision making about resource allocation. The design also allows for the examination of a mediator in CAMS (suicidal cognitions) and mediators in DBT (emotion regulation-based processes and use of skills) in addition to moderators in treatment outcome. The project is further fortified with the incorporation of cost-effectiveness analysis and implementation outcome assessment providing additional data to evaluate the dissemination potential and cost-effectiveness of using these ATSs within a CCC setting. The investigative team is exceptional, with clearly defined roles and a history of collaboration and well-resourced intervention sites. Overall, the resubmission is responsive to the previous review, while some methodological challenges remain. For example, there are challenges to implementing the intervention in a college setting. Specifically, the number of treatment sessions, length of DBT and assessment sessions, and fit with the academic calendar and college demands, thus feasibility and sustainability are in question. Moreover, inadequate consideration of heterogeneity in the TAU group decreases potential generalizability. Finally, there is an insufficient discussion about missing data and treatment response rate. The majority of methodological weaknesses were deemed addressable. The justification for collaboration is sound, but there were questions about the timeline. Upon review, the inclusion plan was deemed acceptable; however, there are concerns about the proposed 48-hour wait time, the protection of EMA data, and potential participant burden. Despite these weaknesses, there is a great deal of enthusiasm for this project, and the investigative team is well poised to conduct such a project.

DESCRIPTION (provided by applicant): Suicide is the 2nd leading cause of death among college students and suicidal ideation and suicide-related behaviors are a frequent presenting problem at college counseling centers (CCCs), which are overburdened. Studies show that some students respond rapidly to treatment, whereas others require considerably more resources. Evidence-based adaptive treatment strategies (ATSs) are needed to address this heterogeneity in responsivity and complexity. ATSs individualize treatment via decision rules specifying how the type and intensity of an intervention can be sequenced based on risk factors, response, or compliance. We are proposing a multisite study to investigate ATSs through a SMART (sequential, multiple assignment, randomized trial) to address suicidal risk in treatment-seeking college students. This multisite study [redacted], submitted in response to RFA- MH-18-700, Collaborative R01s for Clinical Trials, will enroll moderately to severely suicidal college students in the "emerging adulthood" phase (ages 18-25) seeking services at CCCs. This SMART will have two stages of intervention. In the first stage, 700 participants from four CCCs will be randomized to 4-8 weeks of: 1) a suicide-focused treatment – Collaborative Assessment and Management of Suicidality (CAMS) or 2) Treatment as Usual (TAU). Sufficient responders to either intervention will discontinue services/be stepped down. Non- responders will be re-randomized to one of two second-stage higher intensity/dosage intervention options for an additional 4-16 weeks: 1) CAMS (either continued or administered for the first time) or 2) Comprehensive Dialectical Behavior Therapy (DBT), which includes individual therapy, skills groups, and phone/text coaching for the clients and peer consultation for the therapists. The aims of this research project are to 1) compare the efficacy of the four ATSs in

reducing students' suicidal ideation, non-suicidal self-injury, and suicide attempts; 2) evaluate whether, as hypothesized, a sequence that starts with a suicide-focused treatment approach (i.e., CAMS) is more effective in reducing suicidal risk than TAU; 3) determine whether, as hypothesized, a more comprehensive, suicide-focused approach (i.e., DBT) is more helpful as a second stage intervention for insufficient responders, relative to a less intensive suicide-focused approach (i.e., CAMS); 4) assess the mechanisms of change leading to reduced suicidal risk for each treatment; specifically, to evaluate suicidal cognitions as a mediator in CAMS and emotion regulation-based processes and use of skills as mediators in DBT; 5) examine baseline factors as predictors and/or moderators of treatment outcome; 6) evaluate the dissemination potential and cost effectiveness of using these ATSs within a CCC setting. This study will provide essential guidance to CCCs on how to best allocate limited resources to alleviate an increasing public health crisis.

PUBLIC HEALTH RELEVANCE: This multisite study will test an adaptive intervention strategy for college students who are suicidal when seeking treatment at a college counseling center. At this time, the typical strategy relies on a "one-size-fits-all" approach, but in fact suicidal college students vary greatly on what and how much they need. This study will allow clinical decision making (trying one approach, and if that doesn't work, another) to be empirically tested while maximizing resources in overburdened college counseling centers.

CRITIQUE 1

Significance: 1
Investigator(s): 1
Innovation: 1
Approach: 3
Environment: 2

Overall Impact:
This is a resubmission of a collaborative R01 comparing EBTs (CAMS and DBT) in a treatment seeking college population with high suicide risk, using an elegant SMART design in a hybrid efficacy/effectiveness trial. The investigators have been responsive to the majority of the reviewers' comments, including adding data from the pilot study, further justification for intervention choices and the adaptive design, additional outcome measures other than self-report-- including EMA and academic performance/health data. The resubmission includes detail on how clinicians will be trained on EBT and on working with SGM youth, more detail on QA measures and how to reduce contamination effects by clinicians doing all treatments (TAU, CAMS, DBT). They have eliminated two assessment points to reduce participant burden. In addition, cost effectiveness will be examined as well as implementation outcomes. While the application makes a solid case for CAMS as an evidence-based "front line" intervention that is easy to deliver, in the pilot data CAMS did not seem to differ from TAU (CAMS response rate of 48% vs TAU response rate of 44%). In addition, CAMS had a 10% higher attrition rate than TAU, and fared better with less ill participants. The data presented on the RCT with CAMS lacked specifics and more information would be helpful to include. While DBT as a stage 2 intervention was well justified in the application, some indication that this treatment model/intervention can be sustainable in a college setting might strengthen the application. In addition, in the RCT with DBT with college students— no specific data was provided on suicidal outcomes (although they report that DBT reduced suicidality).

1. Significance:
Strengths

- This project addresses a critical public health issue: suicidal ideation and behavior on college campuses. Suicide has been on the rise and has increased in this age group—now the 2nd leading cause of death.
- The design allows for the testing of four adaptive treatment strategies.
- The project will allow for dissemination into other college counseling centers –particularly the CAMS approach which requires fewer resources.

Weaknesses

- None noted.

2. Investigator(s):

Strengths

- Strong team, each with expertise that contributes to the overall study.
- The PIs and their sites have clear roles in the study (oversight of multisite study-[redacted], data management--[redacted], implementation--[redacted], DBT[redacted]).
- There are strong consultants, as well as a scientific advisory board and an identified DSMB.
- The application includes experts in the experimental treatments ([redacted]).
- There is an opportunity for counseling center stakeholder input regarding barriers to implementation and sustainability.
- These investigators have worked together in the past.

Weaknesses

- None noted.

3. Innovation:

Strengths

- The SMART design is innovative.
- Using sequential treatments, stepped design, is also innovative.
- The use of a hybrid effectiveness-implementation multisite study is a strength.
- Randomization strategy includes stratification by gender, past attempts, and medication status.
- Using EMA in this study is also innovative.
- The inclusion of a cost effectiveness analysis is a plus, as well as the implementation outcomes assessments.

Weaknesses

- None noted.

4. Approach:

Strengths

- The overall design takes an effectiveness approach and utilizes a practice-oriented research design.
- Using clinicians at the site to provide the interventions was well justified in the application.
- The design allows a test for four adaptive strategies that are reasonable in this population.
- The treatments tested are specific to suicide treatment and have some empirical support for reducing suicide risk. The PIs have experience with both treatments.
- The multi-site (4) is a plus—allowing for differences in diversity and geographic location.
- The design includes stratification on history of attempts, medication status, and gender.
- The project includes moderator analyses which will provide information that will allow for personalized treatments.
- The application includes mediator analyses to identify mechanisms of change.
- Effectiveness approach is important, as is using existing clinical staff to provide the intervention.
- Instruments used are well selected, well validated, and appropriate for the study.

- Quality control is well described, including an assessment of each intervention to confirm that no elements of each therapy (DBT, CAMS) are contained in comparative treatment conditions.
- The training of the therapists is well described and will be done by experts in each treatment.
- While the primary outcome is suicide risk reduction, the application reports that attempts, and suicidal behaviors will also be measured.

Weaknesses
- While a clear case is made for having all clinicians deliver all treatments, the potential for contamination is a limitation.
- The description of the cost effectiveness and implementation analyses was limited; thus, it was not clear how these will be completed.

5. Environment:
Strengths
- Strong investigative team at each site, with clear capability to conduct the trial.

Weaknesses
- There was only one letter of support from one of the four CCC's ([redacted]. It is not clear that the CCCs at the other three sites in support of conducting an RCT at their center.

Study Timeline:
Strengths
- Timelines and milestones are well specified.

Weaknesses
- None noted.

Collaboration (Only R01 Collaborative applications):
- Adequate.
- There is good justification for a 4-site study--given suicidality has a low base rate, and multiple sites allow for greater diversity and increased ability to disseminate to other settings going forward if findings are positive.

Protections for Human Subjects:
Acceptable Risks and/or Adequate Protections
- Adequate plan for addressing worsening of symptoms and/or suicidal events.
- There could be more information about how the text data from EMA questions is protected/HIPAA compliant.

Data and Safety Monitoring Plan (Applicable for Clinical Trials Only):
- Acceptable.
- The application includes a DSMB (Comtois to chair) will meet prior to trial and twice a year; A Scientific Advisory Board ([redacted] to chair) meeting on same frequency as above.
- A Counseling Center Advisory Board is also included.

Inclusion of Women, Minorities and Children:
- Sex/Gender: Distribution justified scientifically.
- Race/Ethnicity: Distribution justified scientifically.
- For NIH-Defined Phase III trials, Plans for valid design and analysis: Not applicable.
- Inclusion/Exclusion of Children under 18: Excluding ages <18; justified scientifically.

Vertebrate Animals:
Not Applicable (No Vertebrate Animals)

Biohazards:

Not Applicable (No Biohazards)

Resubmission:
- The reviewers were responsive to most comments, either making adjustments or providing further rationale for their decisions.

Renewal:

Revision:

Applications from Foreign Organizations:
Not Applicable (No Foreign Organizations)

Select Agents:
Not Applicable (No Select Agents)

Resource Sharing Plans:
- Unacceptable.

Authentication of Key Biological and/or Chemical Resources:
Not Applicable (No Relevant Resources)

Budget and Period of Support:
Recommend as Requested

CRITIQUE 2

Significance: 1
Investigator(s): 1
Innovation: 2
Approach: 4
Environment: 1

Overall Impact:
This application addresses a critical area of research and targets a population that is under-studied and for whom suicidal thinking and behavior are significant concerns. This multi-site approach has been tested in a successful pilot trial and is put forth by a strong team of investigators that has previously worked together successfully. The SMART design is rigorous and allows for the examination of both mediators and moderators of participants' responses to two different intervention approaches. The stepped approach to care is consistent with the direction that CCCs are moving currently and has the potential to enable CCCs to address the needs of more students in less time. Despite these strengths, enthusiasm for this application is tempered by concerns surrounding its feasibility for implementation within the CCC environment, by concerns about the possibility that assessment burden and the lack of specificity surrounding TAU may interfere with the likelihood of detecting group differences, and by human subjects concerns.

1. Significance:
Strengths
- Suicidal thinking/behavior is a significant public health concern, with rates of suicide increasing among college students.

- Managing suicidal behavior of college students is a priority, and a significant cost, for CCCs – the resources of CCCs are taxed significantly by college students who report suicidal thinking/behavior.
- If a feasible, evidence-based adapted treatment strategy for suicidal risk in college students can be developed and shown to be efficacious across college environments, there will be exciting possibilities regarding disseminating this approach broadly.

Weaknesses

- None noted.

2. Investigator(s):

Strengths

- Members of the team have worked together previously and successfully.
- Roles of team members are clearly outlined.
- The team includes experts in both CAMS and DBT, and these experts will be actively involved in the training of clinicians and the monitoring of intervention fidelity.
- Team members are well-connected to the CCCs at which this study would be implemented and will engage CCC staff as stakeholders.

Weaknesses

- None noted.

3. Innovation:

Strengths

- This is the first example of the use of an adapted treatment strategy targeting suicidal risk in college students.
- The SMART design will enable careful implementation of this adapted treatment strategy.
- Few large-scale clinical trials address the development of treatment strategies specific to the college counseling center environment.

Weaknesses

- None noted.

4. Approach:

Strengths

- The use of the SMART design will allow investigators to determine which combination of treatment approaches is best for college students experiencing SI.
- The use of a multisite team should enable enrollment of a large number of participants, brings together a broad range of expertise, and will provide data on the effectiveness/implementation possibilities of this approach.
- The success of a pilot study reveals that this approach may be feasible within the environment of a CCC.
- The training protocol for therapists seems well-constructed, as is the plan for monitoring adherence to each intervention approach.

Weaknesses

- The assessment schedule, and length of assessments, is quite burdensome. Over the course of a year, participants may spend 16 hours in assessment interviews, in addition to the time they are in treatment.
- The amount of time participants spend in assessments may be equivalent to the amount of time participants spend in intervention, and it is not clear how the assessment interviews differ from the CAMS Suicide Status Form. Assessment benefits (as outlined in the Human Subjects Section – "assessments have been reported as therapeutic by any students") may blur differences between intervention conditions, especially at Stage 1, interfering with the ability to provide evidence supporting one or another intervention.

- It is not clear that this model is feasible within the college setting. For example, the DBT group sessions are two hours long, and it's not clear how that length of time would work with students' schedules. It is also not clear how this much intervention would fit within the college/university academic calendar. If students are bumped into DBT Stage 2 in the midst of a semester, how would they participate in the group program? And how would they complete a course of DBT with a 3-month summer break?
- TAU – not clear what it is, and how it differs from the assessment protocol, and why it is just as acceptable as CAMS, and why it might be better than CAMS for students with more suicide attempts and more features of BPD. It also does not make sense that therapists are not allowed to include strategies from CAMS or DBT in TAU since features from those approaches would likely be part of any effort to address suicidal thoughts/behaviors in clients.

5. Environment:
Strengths
- All four universities have outstanding facilities and resources.
- All sites have expertise needed to complete this project.

Weaknesses
- None noted.

Study Timeline:
Strengths
- The study timeline seems overall feasible.

Weaknesses
- There are some concerns, noted above, about the way in which the intervention approaches will fit within the academic calendar and campus culture, and whether it will be feasible for participants to complete the treatment programs within the schedule outlined.

Collaboration (Only R01 Collaborative applications):
- No concerns.
- Adequate plans for a multisite trial are outlined.

Protections for Human Subjects:
Unacceptable Risks and/or Inadequate Protections
- There is concern about the burden of the assessment process - 16 hours of assessment over a year.
- There doesn't appear to be a plan for addressing safety concerns of participants who miss appointments but at previous appointments were experiencing suicidal thoughts/behaviors. What will be done to encourage these participants to return to treatment, or to monitor their safety, especially if they refuse further assessments?
- When students present with significant SI, 48 hours seems too long for students to wait for a meeting with a clinician at enrollment, or to receive a phone call from a research clinician.

Data and Safety Monitoring Plan (Applicable for Clinical Trials Only):
- Acceptable.
- No concerns

Inclusion of Women, Minorities and Children:
- Sex/Gender: Distribution justified scientifically.
- Race/Ethnicity: Distribution justified scientifically.
- For NIH-Defined Phase III trials, Plans for valid design and analysis: Not applicable.
- Inclusion/Exclusion of Children under 18: Excluding ages <18; justified scientifically.

- The application presents the percentage of students from different racial and ethnic groups at each site, but are there any differences in the rate of accessing CCC services among students from these different racial and ethnic groups?
- Is the enrollment plan realistic given the rates at which students access CCC resources on these campuses?

Vertebrate Animals:
Not Applicable (No Vertebrate Animals)

Biohazards:

Resubmission:
- Comments were generally addressed.

Renewal:

Revision:

Applications from Foreign Organizations:
Not Applicable (No Foreign Organizations)

Select Agents:
Not Applicable (No Select Agents)

Resource Sharing Plans:
- Acceptable.

Authentication of Key Biological and/or Chemical Resources:
Not Applicable (No Relevant Resources)

Budget and Period of Support:
Recommend as Requested

CRITIQUE 3

Significance: 2
Investigator(s): 2
Innovation: 2
Approach: 2
Environment: 2

Overall Impact:
Suicide is a major public health concern at the college level, and suicide ideation and suicide related behaviors frequently present at college counseling centers. Evidence-based adaptive treatment strategies (ATSs) will be compared. This is a large and ambitious study involving multiple sites and a sample of 700 students. There are 4 ATSs, with sequential change in treatment for students considered as non-responders. These approaches will be compared, and mechanisms and moderators will be studied as well. This is a complex study analytically, as well as in implementation. Fidelity of the proposed interventions will be monitored. The treatment as usual arm may be heterogeneous, and is not well characterized, although it will be monitored to avoid mixing of the treatment interventions. The missing data mechanisms will be complex given the school calendar and potentially long treatment

periods with two stages, so the plan for understanding missing data impact on inference could have been elaborated upon more, and the projected attrition rate needs further justification. Overall, the adaptive design and analysis plan are carefully considered. This could be a high impact study.

1. Significance:
Strengths

- Suicide prevention treatment at college counseling centers is a deeply important issue.
- Adaptive and tailored treatments may help alleviate overburdened resources and lead to improved outcomes.

Weaknesses

- [None noted]

2. Investigator(s):
Strengths

- Strong team with solid statistical support.

Weaknesses

- A little concerned that the main statistician is a consultant, and the number of hours allotted for his work may become tight at the end. There are a number of complex analyses to conduct.

3. Innovation:
Strengths

- SMART design with two different suicide prevention therapies in sequence.
- Intervention conducted at college counseling centers.

Weaknesses

- [None noted]

4. Approach:
Strengths

- Carefully crafted SMART design, comprehensive analysis plan to accompany the complex design.
- Multiple college campuses.
- Large sample allows for moderation analysis.
- Cost data will be collected.
- Fidelity to the suicide-specific CAMS and DBT interventions will be monitored.
- Important public health issue.
- This can thus be a high impact study.

Weaknesses

- A concern is with the possible heterogeneity with TAU. TAU may not be the same from site to site, and between therapists. This can affect generalizability of the findings given there are only 4 sites.
- There does not appear to be an attempt to characterize the possible heterogeneity across the TAU services that students will receive, only an effort to minimize mixing with the intervention therapies. It is understood that this is to be an effectiveness trial, but this could still be very helpful in understanding the findings.

- The missing data plan is generic and does not fully acknowledge the complexities of the school setting, such as calendar and break issues, and transfer and graduation rates. Counting sessions with interruption could be problematic, in that it may not be equivalent to same number of sessions administered continuously (dose wise). A lot may have changed during these interruption periods. A solid percentage of students may be graduating seniors, or freshmen that leave the university, etc., and the treatment session can go on for extended periods of time, if a student is a non-responder. The assumed attrition rate may be too low, and more justification might be helpful.
- The methods in Aim 1 do not appear to be adequately specified. It is not clear why combining the Responders in the respective ATS subsamples (which means predominant overlap in subjects between respective pairs of ATS) helps in determining the best approach among the ATS. Aren't effect sizes reduced by the shared data per ATS subsamples? More justification for this approach might have been helpful.
- The power analyses include inputs such as the within subject correlation. More justification for these selections could strengthen the analyses, since these may have an impact on power levels.

5. Environment:
Strengths

- Excellent research sites and settings.

Weaknesses

- [None noted]

Study Timeline:
Strengths

- Recruiting and study timelines seem feasible.

Weaknesses

- [None noted]

Collaboration (Only R01 Collaborative applications):

- Adequate.
- No concerns noted.

Protections for Human Subjects:
Acceptable Risks and/or Adequate Protections

- No concerns noted.

Data and Safety Monitoring Plan (Applicable for Clinical Trials Only):

- Acceptable.
- No concerns noted.

Inclusion of Women, Minorities and Children:

- Sex/Gender: Distribution justified scientifically.
- Race/Ethnicity: Distribution justified scientifically.
- For NIH-Defined Phase III trials, Plans for valid design and analysis:
- Inclusion/Exclusion of Children under 18: Excluding ages <18; justified scientifically.

- No concerns noted.

Vertebrate Animals:
Not Applicable (No Vertebrate Animals)

Biohazards:
Not Applicable (No Biohazards)

Resubmission:

Renewal:

Revision:
- Responsive to many prior concerns, still need better characterization and understanding of TAU, even if this is effectiveness trial

Applications from Foreign Organizations:

Select Agents:

Resource Sharing Plans:

Authentication of Key Biological and/or Chemical Resources:

Budget and Period of Support:
Recommended budget modifications or possible overlap identified:

THE FOLLOWING SECTIONS WERE PREPARED BY THE SCIENTIFIC REVIEW OFFICER TO SUMMARIZE THE OUTCOME OF DISCUSSIONS OF THE REVIEW COMMITTEE, OR REVIEWERS' WRITTEN CRITIQUES, ON THE FOLLOWING ISSUES:

PROTECTION OF HUMAN SUBJECTS: UNACCEPTABLE. Upon review and discussion, the review committee expressed the following concerns with the plan for protection of human subjects: potential participant burden, insufficient plan for addressing safety concerns, extensive wait time if SI identified, and inadequate protection of EMA data.

INCLUSION OF WOMEN PLAN: ACCEPTABLE

INCLUSION OF MINORITIES PLAN: ACCEPTABLE

INCLUSION OF CHILDREN PLAN: ACCEPTABLE

COMMITTEE BUDGET RECOMMENDATIONS: The budget was recommended as requested.

Footnotes for [redacted]

NIH has modified its policy regarding the receipt of resubmissions (amended applications). See Guide Notice NOT-OD-14-074 at http://grants.nih.gov/grants/guide/notice-files/NOT-OD-14-074.html. The impact/priority score is calculated after discussion of an application by averaging the overall scores (1-9) given by all voting reviewers on the committee and

multiplying by 10. The criterion scores are submitted prior to the meeting by the individual reviewers assigned to an application, and are not discussed specifically at the review meeting or calculated into the overall impact score. Some applications also receive a percentile ranking. For details on the review process, see http://grants.nih.gov/grants/peer_review_process.htm#scoring.

MEETING ROSTER

The roster for this review meeting is displayed as an aggregated roster that includes reviewers from multiple MH Special Emphasis Panels of the NIMH Special Emphasis Panel Aggregate Roster for the 2019/05 council round.
This roster for MH is available at:
http://public.era.nih.gov/pubroster/Reports?DOCTYPE=SEP&DESFORMAT=PDF&AGENDA_SEQ_NUM_P=357275
