# Supplementary material for: The Comprehensive Adaptive Multisite Prevention of University Student Suicide Trial: Protocol for a Randomized Controlled Trial
Source: JMIR Res Protoc. 2025 Apr 22;14:e68441. doi: 10.2196/68441 (PMC12056417; doi:10.2196/68441)
Supplement: Multimedia Appendix 2 [file resprot_v14i1e68441_app2.pdf]

June 20, 2019

Adapting Treatments for Suicidal College Students: A Multisite Trial Responses to Reviewers' Comments

Sites/PIs/Award Numbers:
1) [redacted] R01-MH116052-01A1)
2) [redacted] R01-MH116062-01A1)
3) [redacted] R01-MH116050-01A1)
4) [redacted] R01-MH116061-01A1)

We are grateful to the reviewers for their positive review of our proposal (impact score=28), and for the opportunity to respond to the key questions and critiques identified by the reviewers and communicated to us by our Program Officer, Dr. Mary Rooney. Please note that responses related to human subjects matters have been submitted in a separate document. Further, in light of discussions with Dr. Rooney, we have decided to omit the EMA portion of the study. Thus, concerns related to EMA are no longer relevant to the study and this is highlighted when it is applicable.

In addition to our responses below, we want to emphasize that this is a hybrid trial, relying heavily on **effectiveness** and **pragmatic** trial elements, with the goal of identifying an empirically-based, effective sequence of care for suicidal college students that is readily disseminable. To facilitate research translation into practice, more pragmatic approaches for experimental research designs, methods, and measures are necessary (Glasgow, 2013). Pragmatic trials are designed to answer the question of whether intervention strategies work under "real-world" conditions (i.e., effectiveness research), whereas explanatory trials answer the question of whether such strategies work under ideal conditions (i.e., efficacy research). Consistent with the priorities specified in the RFA (MH-18-700), the proposed hybrid effectiveness-implementation SMART design attempts to strike a balance between rigorous controlled experimentation and practice-based research under real-world conditions. As such, some of the design features of the proposed research intentionally lean more toward the pragmatic side of the continuum. The implications of this strategy are that actual dissemination and implementation are more achievable, which will result in more suicidal students receiving evidence-based care, thereby decreasing suicidal suffering and saving lives.

Below, we will outline the concerns relevant to the specific sections of the application.

Significance

CRITIQUE: *Concern about the feasibility of delivering DBT in college counseling centers (CCC). Can you provide data on the number of counseling centers (roughly) that currently offer this intervention?*

RESPONSE: "Comprehensive DBT" (C-DBT; Linehan, 1993) as originally developed was a year-long treatment that, for the client, involved weekly individual therapy sessions and weekly group skills training (two appointments per week for the client). Since its development, DBT has been adapted to fit a wide variety of settings and contexts. These adaptations have included changes to the standard length of treatment, which have included 12 to 16-week adaptations for suicidal adolescents and their families (see Miller, Rathus, & Linehan, 2007) and six-month (24-week) adaptations (e.g., Rizvi, Hughes, Hittman, & Vieira Oliveira, 2017). Although DBT is frequently referred to as an "intensive" treatment (and therefore less feasible to implement), it is important to note that individuals deemed high risk are often offered multiple services per week at CCCs, thus DBT services may not pose any additional burden. C-DBT

delivered at CCCs is often adapted for a shorter period of time [up to 16 weeks in our study (Pistorello & Chugani, in press). Additionally, C-DBT in this study and in general will be used as a Specialty Track within CCCs, thus the provision of more services does not interfere with outstanding policies (such as session limits, etc). Only a small segment of students will need C-DBT, and a less resource-intensive approach [like Collaborative Assessment and Management of Suicidality (CAMS; Jobes, 2012, 2016], can serve as a first-line approach to suicide risk in CCCs. Although there has only been one published RCT with C-DBT at CCCs (Pistorello, Fruzzetti, MacLane, Gallop, & Iverson, 2012), open trials have also been conducted with C-DBT at CCCs (Engle, Gadischke, Roy, & Nunziato, 2013; Panepinto, Uschold, Olandese, & Linn, 2015) and more than a dozen studies have investigated the use of DBT skills groups in CCCs (Chugani & Landes, 2016). **A recent survey concluded that approximately *one third* of CCCs already use some form of DBT** (Chugani & Landes, 2016) and a significant body of research indicates that DBT is effective for the types of problems and level of severity that are routinely treated at CCCs (e.g., Bankoff, Karpel, Forbes, & Pantalone, 2012). Although providing an additional 4-16 sessions in Stage 2 for insufficient responders to Stage 1 treatments might appear to tax CCCs' resources, longer-term care for a segment of the students (20+ sessions) already happens in routine CCC practice (Center for Collegiate Mental Health or CCMH, 2018). Data show that students presenting with threat-to-self already use a third more services (CCMH, 2018) and removing suicidal students from campus may incur increased risk of litigation (Aviv, 2014) as well as eliminate the known and potent protective factor of the campus environment (Lamis & Lester, 2011). However, even for those CCCs that cannot or choose not to implement C-DBT, this study will be informative about the most effective *sequences of care* for suicidal students and will inform which students to refer out to more intensive community approaches, such as C-DBT.

Approach

CRITIQUE: *Concern about the same therapist delivering all three treatments (possible contamination in TAU). There was also a concern about heterogeneity in TAU – and a need to really monitor and characterize TAU.*

RESPONSE: We carefully revisited this option at the time of the resubmission and have concluded that although relying on different therapists for different treatments is desirable from an efficacy-based perspective, there are compelling ethical/clinical, pragmatic, and methodological reasons for having the same therapists deliver all treatments in this study: 1) the option to switch therapists is often provided in studies with high risk populations (Linehan et al., 2006) and it will be offered as an option to participants in this trial as well. However, **requiring** clients with unresolved suicidal risk (and who might struggle with fears of abandonment) to switch therapists at the end of Stage 1 could actually lead to an escalation of suicidal risk (Sansone & Fine, 1991) and/or increased the likelihood of treatment dropout—a worry expressed by members of our Data and Safety Monitoring Board during the pilot study; 2) data show that keeping the same clinician across treatment transitions in a CCC is associated with half as many treatment dropouts and fewer sessions overall (Nielsen, Okiishi, Nielsen, Hawkins, Harmon, Pedersen, et al., 2009); 3) exit interviews in our pilot study revealed that all re-randomized students who stayed with the same therapist in Stage 2 explicitly noted their appreciation for being able to do so; and 4) it is a particularly elegant feature of our study design that providers *serve as their own controls*. Thus, we are removing a significant source of error variance in clinical research (i.e., between-group "therapist factors"), while preserving the effectiveness/pragmatic features of the study that the original RFA

sought. In the real world, clinicians can evolve and/or change their treatment plans without having to refer away a client to another provider with whom the client has no pre-existing relationship (in effect starting over with a new therapist). Within our SMART hybrid experimental design, we nevertheless enhance internal validity by using standardized adherence rating methods (e.g., the CAMS Rating Scale, Corona et al., 2018a; 2018b) to establish between-group fidelity and to ascertain and address concerns about any cross-treatment contamination.

Furthermore, as noted in the application, our pilot findings showed no contamination of CAMS into TAU or DBT and no contamination of DBT into CAMS or TAU. This lack of contamination in TAU has also been supported by other large-scale research (see Santa Ana, Martino, Ball, Nich, Frankforter, & Carroll, 2008). To further reduce the chance that contamination in this trial could occur, however, counselors will be trained on the need to follow the randomly assigned approach and there will be ongoing fidelity checks and feedback given to counselors on all conditions throughout the course of the trial (something that was not done during the pilot because adherence/contamination was viewed as a DV in itself in that case and only assessed at the end). Thus, even without this ongoing feedback, there was no contamination in the pilot; the provision of ongoing monitoring, as proposed here, will further offset the potential for contamination. Furthermore, as was done in the pilot, CAMS and DBT cases will be discussed in separate supervision teams and TAU cases at staff meetings (as routinely done in CCCs) to further monitor and reduce any contamination.

We considered optimizing TAU in some way (as done in the original DBT study with college students—Pistorello et al., 2012), in order to make it more uniform, but again, our focus on effectiveness suggests that this would not be wise. Treatment provision at CCCs varies considerably and this reflects the reality of the setting. However, taking reviewers' concerns into consideration, we will collect more information from counselors both prior to training and subsequently on which theoretical orientation they plan to use and later did use in TAU, and as noted above, give them ongoing feedback on their CAMS or DBT adherence ratings of TAU sessions. Moreover, because we are viewing sessions for adherence and fidelity purposes, similar to what has been done in other studies (e.g., Santa Ana et al., 2008), we will explore ways to characterize the treatments being used to have a more informed sense of what TAU for the treatment of suicidal risk actually was in the study and to evaluate if TAU changes across time within a given therapist or if the TAU delivery is impacted by various therapist factors (e.g., demographics, theoretical orientation). This could in fact be an ancillary study within this large-scale trial that would be significant to the field of suicide prevention on its own: What do clinicians do to address suicidal risk when not utilizing structured approaches like CAMS or DBT?

CRITIQUE: *Concern about the intervention length/intensity and fit with a college calendar.*

RESPONSE: We agree with the reviewers that studying interventions that may straddle academic terms is messy. Again, the effectiveness elements of this real-world study "win out" against tight controls that would increase internal validity but render results much less generalizable and potentially more difficult to disseminate because the treatments would not map on to the reality of existing CCC care. Treatment of suicidal college students, even outside of CCCs, inherently involves these challenges (even a private practitioner seeing a college student would have to resolve what to do when a suicidal student goes home during breaks). This is exactly why a study with suicidal college students seeking services at a CCC is critically needed. CCCs all over the country are

doing this work, in whatever form possible, with little guidance. However, in our experience, researchers often shy away from conducting clinical research with this population in part because of these very real-world challenges. Ironically, college students have historically often been a convenience sample (through introductory psychology subject pools), but not as research participants in their own right and for their own benefit. The investigative team has considerable experience conducting clinical research with college students and have thoughtfully developed accommodations to the academic setting. For example, CAMS and DBT individual sessions in this study may be conducted over the phone or via HIPAA-compliant video-conferencing during breaks, depending on counselor and participant preference and clinical need. The end of an academic term often serves as a natural break in treatment in CCCs. On a case-by-case basis, participants may take a “break” from the study treatment while they are on short academic breaks and then continue where they left off upon return. This is an essential adaptation to the setting and it has been considered in terms of recruitment rates and will also be considered in the analyses (e.g., dosage defined as number of sessions received and not time since randomization per stage). Suicide is the second leading cause of death on campus and RCTs in CCC environments are critically necessary despite the difficulty of the college calendar.

Data Analysis

CRITIQUE: *Concern about attrition – how will attrition be handled in terms of data collection and analysis.*

RESPONSE: As described in the Statistical Design and Power section of the application, we intend to conduct intent-to-treat analyses to address the study aims. We have estimated that 20% of the sample may have missing outcomes due to dropout and have accounted for study attrition in the power calculations. To minimize missing data during data collection, we propose to compensate the participants commensurate with the assessment burden for each of the assessments ($10 to $40 per assessment—not a trivial amount for many college students). We will also include reminder and follow-up messages to minimize missing data. In addition, we plan to collect “permanent” contact information at baseline (e.g., family member or close friend) to facilitate data collection with students who may graduate, drop out of school, or who otherwise cannot be contacted based on their limited personal contact information (e.g., residence hall address). Students who have left school (e.g., dropout, graduation) can be assessed via phone calls and/or HIPAA-compliant videoconference (particularly at follow-up). In this sense, although a student might be considered a “treatment drop-out” they are not viewed as a “study drop-out” as they will continue to provide outcome data.

Statistically, we plan to conduct missing data analyses to investigate the potential mechanisms for missingness. Missing data will be dealt with explicitly using multiple imputation (MI) procedures. In stability analyses, data for all aims will be analyzed with and without the MI strategy. Any discrepancies will be reported and carefully examined. We anticipate that nonresponse may depend on unobserved factors; that is, we assume a not missing at random (NMAR) missingness mechanism. Therefore, we will also consider joint longitudinal mixed-effects models of suicide risk scores and response status. We will also consider fitting pattern mixture and selection models which allow for an NMAR missingness mechanism. As a result of using these methods, all randomized intent-to-treat subjects will be included in analyses, and over-recruitment to account for loss to follow-up will not be necessary.

CRITIQUE: *Concern about lack of analytic plan for EMA data*.

RESPONSE: In light of the removal of the EMA portion of the proposal, this critique is no longer relevant.

References

Aviv R. (2014). Should suicidal students be forced to leave campus? *The New Yorker*; News Desk.

Bankoff, S. M., Karpel, M. G., Forbes, H. E., & Pantalone, D. W. (2012). A systematic review of dialectical behavior therapy for the treatment of eating disorders. *Eating Disorders, 20*(3), 196-215. doi:10.1080/10640266.2012.668478

Center for Collegiate Mental Health. (2018, January). 2017 Annual Report (Publication No. STA 18-166).

Corona, C.D., Gutierrez, P.M., Wagner, B. M., & Jobes, D. A. (2018a). The psychometric properties of the Collaborative Assessment and Management of Suicidality rating scale. *Journal of Clinical Psychology*, 1–12. https://doi.org/10.1002/jclp.22699

Corona, C. D., Gutierrez, P. M., Wagner, B. M., & Jobes, D. A. (2018b). Assessing the reliability of the CAMS rating scale using a generalizability study. *Crisis: The Journal of Crisis Intervention and Suicide Prevention.* https://doi.org/10.1027/0227-5910/a00065

Chugani, C.D. & Landes, S.J. (2016). Dialectical behavior therapy in college counseling centers: Current trends and barriers to implementation. *Journal of College Student Psychotherapy, 30*(3), 176-186.

Engle, E., Gadischkie, S., Roy, N., & Nunziato, D. (2013). Dialectical behavior therapy for a college population: Applications at Sarah Lawrence College and beyond. *Journal of College Student Psychotherapy*, 27(1), 11–30. doi:10.1080/87568225.2013.739014

Glasgow, R. E. (2013) What does it mean to be pragmatic? Pragmatic methods, measures, and models to facilitate research translation. *Health Education & Behavior, 40(3)*, 257-265.

Jobes, D. A. (2012). The collaborative assessment and management of suicidality (CAMS): An evolving evidence-based clinical approach to suicidal risk. *Suicide and Life-Threatening Behavior, 42,* 640-653.

Jobes, D. A. (2016). *Managing suicidal risk: A collaborative approach 2nd edition.* New York: The Guilford Press.

Lamis, D. A. & Lester, D. (Eds.) (2011). *Understanding and preventing college student suicide.* Springfield, IL: Charles.C. Thomas.

Linehan, M.M. (1993). *Cognitive behavioral therapy of borderline personality disorder.* New York: Guilford Press.

Linehan, M. M., Comtois, K. A., Murray, A. M., Brown, M. Z., Gallop, R. J., Heard, H. L., . . . Lindenboim, N. (2006). Two-year randomized controlled trial and follow-up of dialectical behavior therapy vs. therapy by experts for suicidal behaviors and borderline personality disorder. *Archives of General Psychiatry, 63*, 757–766. doi:10.1001/archpsyc.63.7.757

Miller, A.L., Rathus, J.H., & Linehan, M.M. (2007). *Dialectical behavior therapy with suicidal adolescents.* New York: Guilford Press.

Nielsen, S. L., Okiishi, J., Nielsen, D. L., Hawkins, E. J., Harmon, S. C., Pedersen, T., …Jackson, A. P. (2009). Termination, appointment use, and outcome patterns associated with intake therapist discontinuity. *Professional Psychology: Research and Practice, 40*(3), 272-278.

Panepinto, A.R., Uschold, C.C., Oldanese, M., & Linn, B.K. (2015). Beyond borderline personality disorder: Dialectical behavior therapy in a college counseling center. *Journal of College Student Psychotherapy, 29*, 211-226.

Pistorello, J. & Chugani, C. (In Press). Dialectical Behavior Therapy (DBT) in university counseling centers (UCCs). Chapter to appear in L. A. Dimeff, S. L. Rizvi, and K. Koerner (Eds), *Dialectical Behavior Therapy in Clinical Practice, Second edition*. New York, NY: Guilford Press.
Pistorello, J., Fruzzetti, A. E., MacLane, C., Gallop, R., & Iverson, K. M. (2012). Dialectical behavior therapy (DBT) applied to college students: A randomized clinical trial. *Journal of Consulting and Clinical Psychology*, *80(6),* 982-994.
Rizvi, S. L., Hughes, C. D., Hittman, A. D., & Vieira Oliveira, P. (2017). Can trainees effectively deliver dialectical behavior therapy for individuals with borderline personality disorder? Outcomes from a training clinic. *Journal of Clinical Psychology, 73*(12), 1599-1611.
Sansone, R. A., & Fine, M.A. (1991). Treatment impressions and termination experiences with borderline patients. *American Journal of Psychotherapy*, *4*(2), 173.
Santa Ana, E. J., Martino, S., Ball, S. A., Nich, C., Frankforter, T. L., & Carroll, K. M. (2008). What is usual about "treatment-as-usual"? Data from two multisite effectiveness trials. *Journal of Substance Abuse Treatment*, *35*(4), 369-379.

[redacted]
