# Supplementary material for: The Comprehensive Adaptive Multisite Prevention of University Student Suicide Trial: Protocol for a Randomized Controlled Trial
Source: JMIR Res Protoc. 2025 Apr 22;14:e68441. doi: 10.2196/68441 (PMC12056417; doi:10.2196/68441)
Supplement: Multimedia Appendix 3 [file resprot_v14i1e68441_app3.docx]

# **The Comprehensive Adaptive Multisite Prevention of University student Suicide (CAMPUS) Trial: Protocol for a Randomized Controlled Trial**

## **Author commentary on original peer-review reports from the granting agency**

The protocol presented in this publication differs from the initial design that was peer-reviewed by our funding agency. These modifications were necessary due to the unforeseen impact of the COVID-19 pandemic, which posed challenges that could not have been anticipated at the time of initial planning. To ensure participant safety and adapt to logistical constraints, we implemented substantial changes in the trial design and methodology.

These modifications were carefully considered and implemented in consultation with our scientific advisory board, university counseling center implementation advisory board, and program officer from the funding agency. Each of these stakeholders provided invaluable guidance to ensure that the changes maintained the scientific integrity of the study, while allowing us to adapt to the evolving situation. All modifications were designed to mitigate potential biases that could arise from the altered circumstances. Any adjustments to the initial design were transparently documented and reviewed by the funding agency, the institutional review boards, and our Data Safety Monitoring Board. This approach aims to ensure that the study findings remain as unbiased as possible, despite the external constraints encountered.
